# Mitomycin Gel for the Pyelocaliceal System in Patients With Urinary Diversion: First Reported Cases and Operative Technique

**DOI:** 10.1155/criu/2901637

**Published:** 2026-04-06

**Authors:** Kai Wen Cheng, Grant Sajdak, Ala′a Farkouh, Sikai Song, Brian Hu

**Affiliations:** ^1^ Department of Urology, Loma Linda University Medical Center, Loma Linda, California, USA, lomalindahealth.org; ^2^ School of Medicine, Loma Linda University, Loma Linda, California, USA, llu.edu

## Abstract

**Purpose:**

Mitomycin gel for the pyelocaliceal system (UGN‐101) is an approved kidney‐sparing therapy for upper tract urothelial carcinoma (UTUC). Little to no data exist on its safety and efficacy in patients with a history of urinary diversion. We report our experience with overcoming the anatomical challenges of UGN‐101 instillation in patients with UTUC and prior history of radical cystectomy with urinary diversion.

**Materials and Methods:**

Retrospective review was performed on all patients with pre‐existing urinary diversion who underwent UGN‐101 instillation for management of UTUC at a single academic institution. Surgical techniques for instillation are described, and the clinical course, including complications and disease status, are reported.

**Results:**

Two patients met inclusion criteria. One patient had carcinoma in situ in the renal pelvis and was treated with antegrade instillation via nephrostomy tube at an infusion clinic. The second patient had multifocal disease in the renal pelvis and the distal ureter and was treated with targeted antegrade instillation using an end‐hole catheter. Both patients experienced infectious complications, and one had a ureteral stricture that resolved without intervention. No diversion‐related complications were observed. Both patients are disease‐free at last follow‐up (10 months) on endoscopy and biopsy, although one patient required systemic immunotherapy.

**Conclusions:**

Our series highlights the potential role of UGN‐101 in patients with UTUC and pre‐existing urinary diversion. Antegrade instillation of UGN‐101 can be tailored to specific disease locations and should be considered for patients with a history of urinary diversion who are not candidates for extirpative surgery.

## 1. Introduction

Upper tract urothelial carcinoma (UTUC) presents a clinical challenge due to its development in patients of advanced age, inaccuracies in clinical staging, diverse treatment options, and high recurrence rates with endoscopic treatment. Low‐risk tumors are often ablated endoscopically. In patients with high‐risk UTUC, radical nephroureterectomy can be curative but may be contraindicated in patients with comorbidities or compromised renal function. A newer approach with chemoablation has emerged with mitomycin for the pyelocaliceal system (UGN‐101, Jelmyto), which is administered topically. Shortly after instillation, UGN‐101transforms from its liquid form into a gel within the collecting system at body temperature. This allows for a prolonged dwell time of 4–6 h to amplify the therapeutic effect of mitomycin. UGN‐101was approved as a kidney‐sparing therapy for low‐grade UTUC and has been incorporated into guideline‐based management [1, 2].

In patients receiving UGN‐101, urinary anatomy and the method of administration play a fundamental role. Due to the field effect of urothelial cancer, those with UTUC can have synchronous or metachronous bladder cancer. Patients diagnosed with UTUC who have a history of cystectomy with urinary diversion present a unique challenge for nephron‐sparing treatments. Difficult retrograde access to the ureteral orifices, damage to the urinary diversion, and systemic absorption of chemotherapy represent potential complications. The risk of ureteral stricture, a complication seen with UGN‐101 [3], can be particularly detrimental to patients who have a urinary diversion. Some of these concerns may be mitigated by antegrade administration with early data demonstrating lower rates of stricture [4].

This case series describes the clinical course for the two patients with ileal conduit urinary diversion who received UGN‐101 for UTUC at our institution since the introduction of this treatment modality. To our knowledge, this is the first published report examining the instillation techniques as well as the safety and efficacy of UGN‐101 in patients with pre‐existing urinary diversion.

## 2. Case Report

### 2.1. Case 1

An 89‐year‐old man initially diagnosed with cT3N0M0 urothelial carcinoma (UC) of the bladder was managed with neoadjuvant chemotherapy and robotic radical cystoprostatectomy with intracorporeal ileal conduit urinary diversion. Pathology revealed no residual carcinoma (ypT0) of his bladder and skip lesions of the right ureter with a positive margin for carcinoma in situ (CIS). The patient was placed on surveillance. One and a half year after cystectomy, he presented with gross hematuria and acute kidney injury. Imaging demonstrated right perinephric stranding and blood in the renal pelvis. Right retrograde pyelography demonstrated filling defects in the calyces and renal pelvis. Endoscopy showed blood clots and white material in the renal pelvis without clear tumors. Biopsy and cytology of the renal pelvis were consistent with CIS.

With shared decision‐making, given the patient′s chronic kidney disease (eGFR 35 mL/min/1.73m^2^) and ECOG performance status of 2, we proceeded with UGN‐101 administration via antegrade instillation. The patient was not a good candidate for radical nephroureterectomy, and endoscopic ablation would have been limited without a discrete tumor.

The patient underwent nephrostomy tube insertion with renal pelvic volume measured at 7 mL via antegrade pyelography. Two weeks later, he began weekly antegrade UGN‐101 instillations via his nephrostomy tube at an outpatient infusion center. Figure 1 illustrates the instillation technique. A total of 7 mL of UGN‐101 was injected followed by a 2‐mL normal saline flush. To improve the dwell time of UGN‐101, the nephrostomy was left to gravity for 3 h as this minimizes antegrade urine flow, which may wash out the therapeutic agent. The patient was discharged with instructions to keep the nephrostomy tube capped.

Figure 1Antegrade instillation of mitomycin gel via nephrostomy. (a) Instillation of volume consistent with prior renal pelvic volume measurements with nephrostomy tube left to gravity. (b) Three h after instillation the nephrostomy is capped and facilitates antegrade passage of degraded mitomycin gel.

**Figure:**
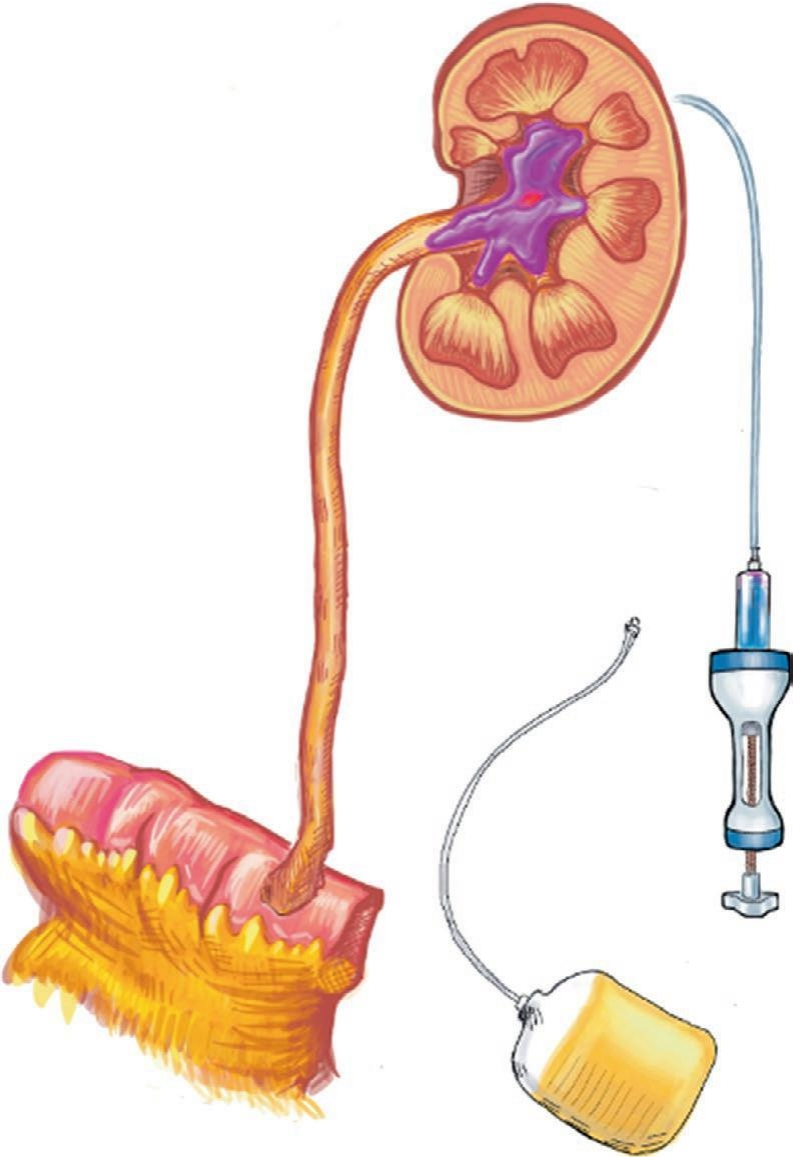
(a)

**Figure:**
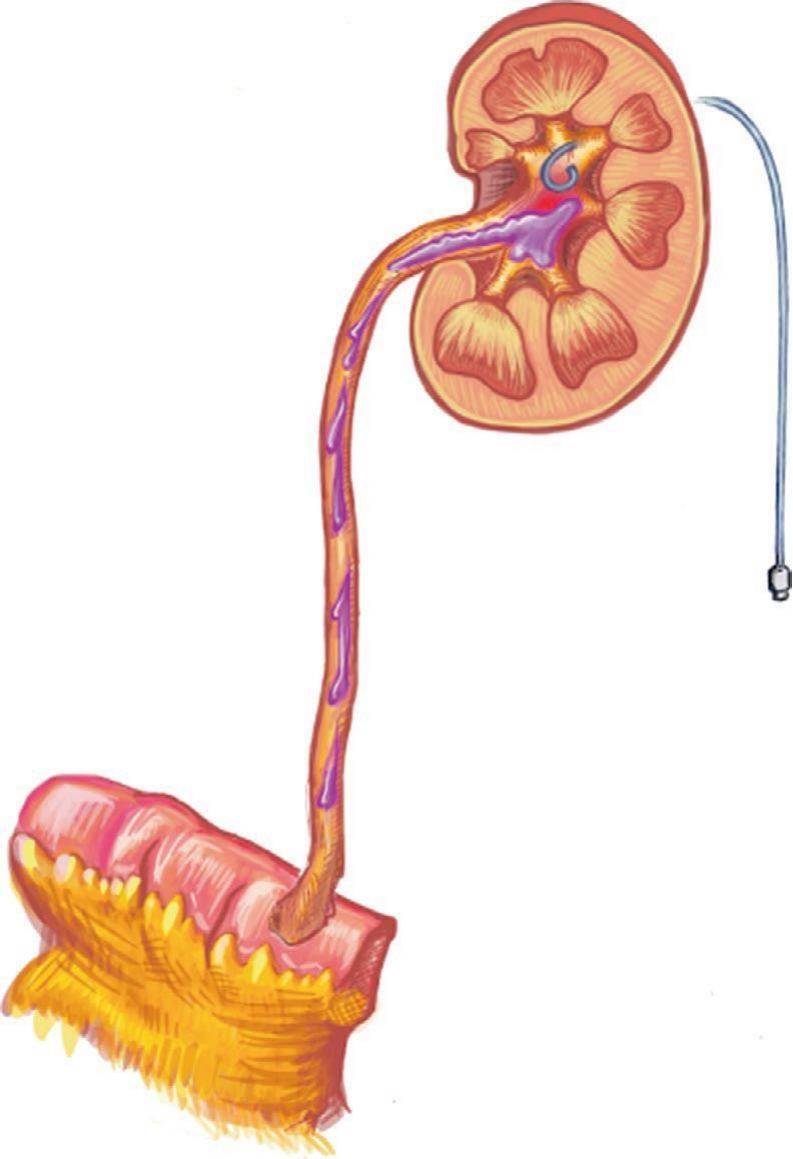
(b)

After his fourth instillation, he developed gross hematuria via his ileal conduit. Looposcopy revealed a new nodular tumor at the right ureteroenteric anastomosis. The tumor was ablated, and biopsy was consistent with high‐grade UC. With progression of disease after four of six planned UGN‐101 instillations, the patient was discussed in a multidisciplinary fashion. As he was unfit for radical surgery and systemic chemotherapy, we proceeded with trimodality therapy, including endoscopic ablation, completion of UGN‐101 instillations, and pembrolizumab. After receiving his fifth instillation of UGN‐101 and first dose of pembrolizumab, he was hospitalized for treatment of multidrug resistant UTI, and his final UGN‐101 instillation was deferred.

Two months after the fifth UGN‐101 instillation, he underwent retrograde ureteroscopy. There were no tumors. Erythematous areas were biopsied. There was concern for pathologic artifact related to UGN‐101 instillation, but pathology favored CIS. He received pembrolizumab for a total of 4 months. Repeat retrograde ureteroscopy with biopsy 10 months after completing UGN‐101 was negative for malignancy. No diversion‐related complications from UGN‐101 administration were noted.

### 2.2. Case 2

An 86‐year‐old man with cT1N0M0 UC of the bladder underwent robotic radical cystoprostatectomy with intracorporeal ileal conduit urinary diversion. Intraoperative frozen sections from the right ureter were positive for CIS. Final pathology revealed pTisN0M0 UC of the bladder and pT1N0M0 right distal ureter UC with negative margins. He was placed on surveillance. One year after surgery, he presented with hematuria and CT evidence of right hydronephrosis and ureteral thickening concerning for UTUC. Retrograde access to the right collecting system was unsuccessful. He underwent right antegrade ureteroscopy. Tissue biopsied from the distal ureter just proximal to the anastomosis confirmed high‐grade UC, and barbotage cytology from an erythematous right renal pelvic lesion was suspicious for UC.

Management was challenging given significant comorbidities including a history of myocardial infarction, atrial fibrillation on anticoagulation, Type II diabetes mellitus, and Stage III chronic kidney disease. With shared decision‐making, we proceeded with UGN‐101 instillations. To maximize dwell time on the urothelium, the plan was made to initiate administration of UGN‐101 at the affected ureteral segment and some in the renal pelvis where barbotage was positive. Antegrade instillations were performed in the operating room under sedation.

Figure 2 illustrates the antegrade instillation for this patient. A 7‐Fr open‐ended ureteral catheter was placed at the ureteroenteric anastomosis just distal to the ureteral UC. UGN‐101 was injected gradually into the right ureter as the 7‐Fr catheter was retracted back to the ureteropelvic junction where the remainder of UGN‐101 was instilled. A new nephrostomy was inserted, placed to gravity drainage for 3 h, and subsequently capped until the next instillation.

Figure 2Antegrade instillation of mitomycin gel via end‐hole catheter. (a) Start of instillation with a 7‐Fr open‐ended ureteral catheter positioned at the ureteroenteric anastomosis, then slowly retracted while instilling. (b) End of instillation with the open‐ended catheter now at the renal pelvis.

**Figure:**
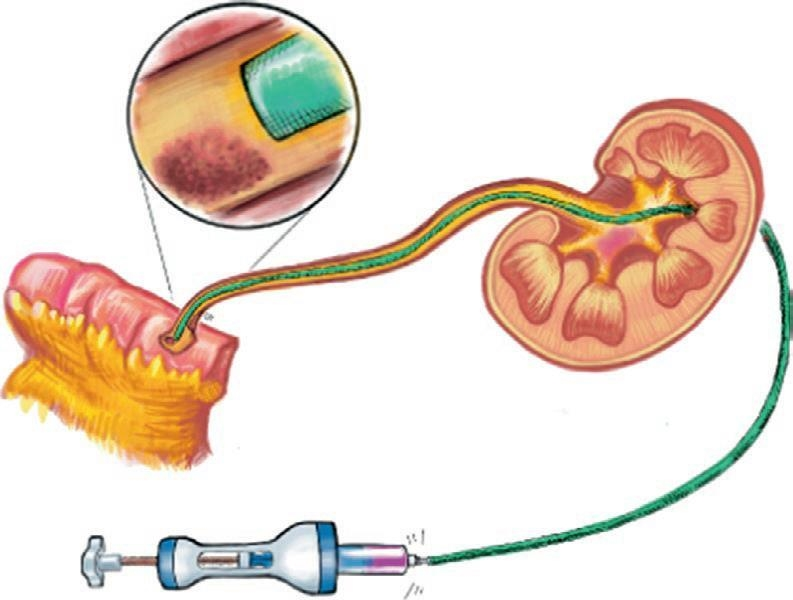
(a)

**Figure:**
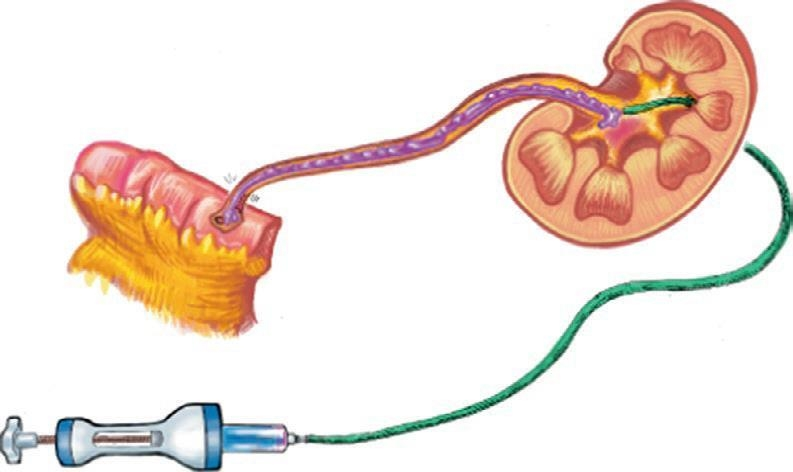
(b)

The patient underwent a total of 5‐weekly instillations in the same manner as described. On the last antegrade pyelogram, a proximal ureteral narrowing was noted (Figure 3a). To mitigate the risk of ureteral stricture, the sixth cycle was aborted. Two months later, loopogram demonstrated reflux bilaterally and right hydroureteronephrosis with no stricture or filling defect (Figure 3b). Looposcopy was normal and retrograde ureteroscopy demonstrated mild inflammatory changes in the ureter without strictures. Cytology and biopsies from the ureter and the renal pelvis were negative. Postoperative course was complicated by UTI, which resolved with antibiotic treatment. There were no diversion‐related complications and renal function remained stable. At 10 months from UGN‐101, axial imaging and repeat ureteroscopy with biopsy were negative for malignancy.

Figure 3Ureterograms during and after treatment. (a) Narrowing during instillations (prone) and (b) surveillance loopogram with right reflux with resolution of narrowing (supine).

**Figure:**
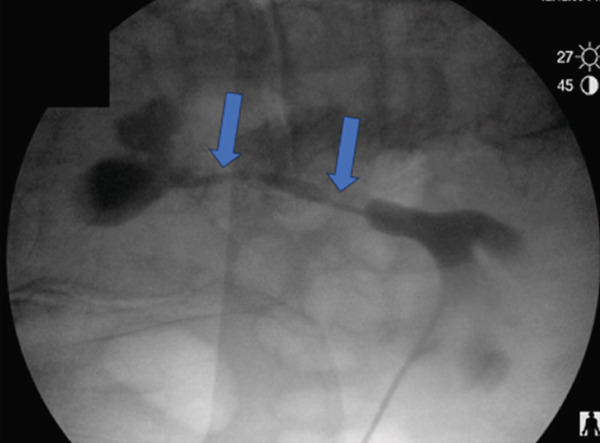
(a)

**Figure:**
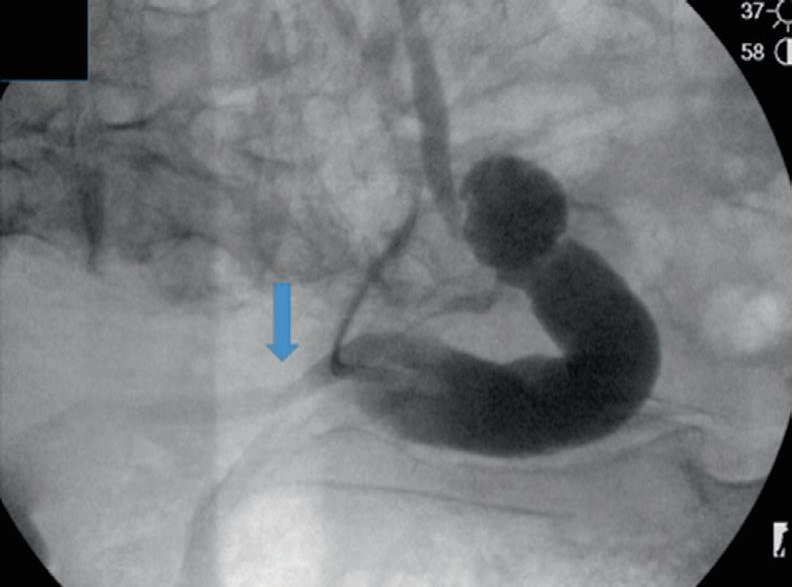
(b)

## 3. Discussion

Treatment of low‐grade UTUC in the pyelocaliceal system with UGN‐101 was approved as a kidney‐sparing modality. However, it is possible that patients with UTUC in the ureter or high‐grade disease could benefit from off‐label use of this therapy, especially in the setting of significant comorbidities limiting extirpative surgery. This series illustrates the complexity of managing patients with UTUC. Both patients described were octogenarians with significant comorbidities, compromised renal function, and multifocal UTUC diagnosed at the time of cystectomy. As such, medical decision‐making is intricate and best done with shared decision‐making with respect for patient‐oriented goals [5]. The decision for nephroureterectomy can be difficult due to a high complication rate (38%–44%) with a significant decrease in eGFR exacerbated by increased age [6, 7]. One recent study by Rose et al. included 12 patients with high‐grade UTUC (Ta, T1 disease) who underwent UGN‐101 instillation due to comorbidities precluding extirpative therapy [8]. In this cohort, 11 patients underwent primary disease evaluation after induction therapy, and five patients (45%) had no evidence of disease, whereas the remainder six patients (55%) had reduced tumor burden. Of the five patients with complete response, two patients developed recurrence after a median of 8.4 months. Although the efficacy of kidney‐sparing modalities in the setting of high‐grade UTUC are not well defined, these treatments may offer acceptable disease control with reasonable preservation of quality of life in select patients with high‐grade disease.

In particular, UGN‐101 is administered with a novel design to prolong dwell time of the therapeutic agent and may offer benefit over other intraluminal therapies for UTUC. Utilizing this property can play a critical role in treatment success by optimizing urothelium contact time, especially in challenging disease locations that have limited meaningful targeted administration of other intraluminal therapies. As some patients with UTUC will have had a urinary diversion, understanding the associated potential side effects remains important. Most notably, systemic absorption, ureteral strictures, and diversion‐related complications can be detrimental events for this patient population. Thus, improving techniques for safe and effective antegrade instillation of UGN‐101 is a promising target to minimize these complications and possibly optimize oncologic outcomes.

Our series demonstrates that antegrade UGN‐101 instillation in select patients with pre‐existing urinary diversion is feasible. For our first patient, UGN‐101 was delivered via nephrostomy tube in an outpatient setting. He required immunotherapy for equivocal histology favoring UC 2 months after UGN‐101 but was disease‐free at 10 months follow‐up. The second patient was treated by targeted antegrade instillation by using a ureteral catheter to direct UGN‐101 at his ureteral UTUC. This patient had a complete response. Both experienced complications of infection and temporary renal dysfunction. One patient had a temporary stricture, which resolved without intervention. These complications are in line with previous studies in patients receiving UGN‐101 with intact lower tract anatomy [3]. Neither patient experienced a diversion‐related complication. Potential side effects of ureteroenteric narrowing, conduit mucosal inflammation, or systemic absorption of mitomycin were not seen.

## 4. Conclusion

The safety and efficacy of UGN‐101 in patients with UTUC and pre‐existing urinary diversion have not been well‐reported. Our series highlights two patients with high‐grade, multifocal UTUC who are older with significant comorbidities that limited extirpation. In these patients, antegrade instillation of UGN‐101 was tailored to cancer location, and no diversion‐specific complications were observed. UGN‐101 was successful in treating the UTUC with a durable response, although one patient required systemic immunotherapy. Longitudinal follow‐up in a larger cohort is needed to further evaluate the safety and efficacy of targeted antegrade instillation of UGN‐101 and should include patients with high‐grade disease, other forms of urinary diversions, and varying disease locations.

## Author Contributions

Brian Hu had full access to all of the data in this study and takes complete responsibility for the integrity of the data and the accuracy of the data analysis.

## Funding

No funding was received for this manuscript.

## Disclosure

All authors have read and approved the final version of the manuscript.

## Consent

No written consent has been obtained from the patients as there is no patient identifiable data included in this case report.

## Conflicts of Interest

Brian Hu is a consultant for Urogen. The other authors declare no conflicts of interest.

## Data Availability

The data that support the findings of this study are available on request from the corresponding author. The data are not publicly available due to privacy or ethical restrictions.
